# Model of the Reticular Formation of the Brainstem Based on Glial–Neuronal Interactions

**DOI:** 10.1007/s12559-014-9260-5

**Published:** 2014-06-03

**Authors:** Bernhard J. Mitterauer

**Affiliations:** Volitronics-Institute for Basic Research, Psychopathology and Brain Philosophy, Gotthard Guenther Archives, Autobahnweg 7, 5071 Wals/Salzburg, Austria

**Keywords:** Reticular formation, Model, Glial–neuronal interactions, Glial network, Computation

## Abstract

A new model of the reticular formation of the brainstem is proposed. It refers to the neuronal and glial cell systems. Thus, it is biomimetically founded. The reticular formation generates modes of behavior (sleeping, eating, etc.) and commands all behavior according to the most appropriate environmental information. The reticular formation works on an abductive logic and is dominated by a redundancy of potential command. Formally, a special mode of behavior is represented by a comprehensive cycle (Hamilton loop) located in the glial network (syncytium) and embodied in gap junctional plaques. Whereas for the neuronal network of the reticular formation, a computer simulation has already been presented; here, the necessary devices for computation in the whole network are outlined.

## Introduction and Hypothesis

A model of synaptic information processing based on glial–neuronal interactions has already been published in this journal [1]. Here, I attempt to elaborate this model for the glial–neuronal interactions in the reticular formation of the brainstem. Whereas the anatomical structure of the neuronal system in the reticular formation has already been identified [2, 3], the glial network is as yet unknown. It is only certain that astrocytes do occur in this system [4].

My hypothetical model is as follows: Since astrocytes determine the function of the neuronal system in the reticular formation, astrocytes must be interconnected via gap junctions building a network, called syncytium. As already hypothesized [5], the glial syncytium may generate intentional programs whose realization is dependent on information from the neuronal system computed from the inner and outer environment. In the case of the reticular formation, the neuronal system computes so-called modes of behavior (eating, sleeping, working, etc.) which must be rapidly generated dependent on the environmental situation. These may guarantee the maintenance of the elementary organization of a living system.

The applied formalism uses exchange relations between neighbored values in the sense of permutations in an *n*-valued system. This allows the generation of integrative circles that comprise all values once, so-called Hamilton loops. The neuronal system in the reticular formation may be comparable to a stack of poker chips, each embodying a Hamilton circle. The glial syncytium builds plaques of gap junctions. Each plaque may embody all necessary gap junctional channels for generating Hamilton loops. These genetically or environmentally determined intentional programs command the neuronal system as to which Hamilton loop is to be selected in correspondence to the behavioral mode. In a robot brain, these double functions can be implemented as a command and an executive computer. Jellema and coworkers [6] have proposed a perception system working according to an abductive logic. This system can be implemented in a robot brain. With concern to our graph-theoretical formal approach to a simulation of the reticular formation, Humphries et al. [7] have developed a formal model of the reticular formation that is comparable to our model (permutographs), but it does not refer to the glial system.

## The Concept of the Modes of Behavior

According to Iberall and McCulloch [8], a living system like man is highly dynamic. In order to produce an integrated behavior, it must be capable of generating stable system states, the so-called modes of behavior. This concept has been somewhat neglected in Brain and Behavioral Sciences, whereas it adopts a pivotal role in the brain model presented here. We do not normally think of human behavior as modal, though most people would agree that their quality of consciousness is unitary and they can only do one thing well at a time [9]. This may be identified as a dynamic action mode of the system, such as “the system sleeps”. In Table 1, the essential modes of behavior or action modes are listed which will have a time constant of the order of the female menstrual period. Although the list itself could be questioned, we would like to focus on the exploratory power of this scientific approach.

**Table 1:** Frequencies of the modes of behavior within about a 4-week cycle (Iberall and McCulloch [8])

Modes of behavior	Percent of time
Sleeps	30
Eats	5
Drinks	1
Voids	1
Sexes	3
Works	25
Rests (no motor activity, indifferent internal sensory activity)	3
Talks	5
Attends (indifferent motor activity, involved sensory activity)	4
Motor practices (runs, walks, plays, etc.)	4
Angers	1
Escapes (negligible motor and sensory input	1
“Anxious-es”	2
“Euphorics”	2
Laughs	1
Aggresses	1
Fears, fights, flights	1
Interpersonal attends (body, verbal or sensory contact)	8
Envies	1
Greeds	1
Total	100 ± 20 % of time involvement

McCulloch [9] has associated the ability of the brain to integrate its functions with the reticular formation in the brain stem, in the sense of an “integrative matrix” [2, 3]. Over time, however, the reticular formation seems to have attracted the interest of scientists in its role as an activating or arousal system. In the 1980s, we further elaborated McCulloch’s theory of reticular formation [10]. The actual molecular enlightenment of the circadian and ultradian oscillators (rhythms) as well as the undeniable influence with which the glial system acts on the neuronal system is a challenge to reconsider the integrative decision function of the reticular formation using the principles of musical composition as a paradigm.

The reticular formation operates by an abductive logic [10–13]. Abduction is the selection of the appropriate program from a repertoire in accordance with a rule for analyzing program requests. These programs are general in the sense that all are principally adapted for the processing of environment information; however, at the same time, they are highly specialized for the processing of specific environment information. When specific environment information acts on the system, the system can decide or select to which program the information belongs, that means, which program is best suited for information processing. The repertoire of these programs represents a heterarchic system (circular system) which is equipped with a “redundancy of potential command” [14], because every program in itself is capable of ruling the whole system for a certain time. When this abductive selection and commanding system are transferred to our brain model, a glial–neuronal compartment corresponds to one respective program structure. These program structures are genetically determined, and the activity of the programs alters with different timescales. Therefore, the brain permanently operates in different system states which correspond not only genetically but also in relation to the environment and to intentions [15]. These program structures or compartments may also be regarded as hypotheses or intentions which are tested in the environment. Since conditions in the environment can quickly change or remain unchanged, the brain must either change its multi-compartmental program structure or “freeze” the biorhythm on a determined program structure. In any case, the program structure that best suits the environment information will command. Compartments in which the environment information does not fit will be “switched off” or rejected temporarily. As it seems to be not only a question of the synchronization of the functions of the total system but also of a spatiotemporal structuring in relation to the environment, the term harmonization could be justified.

## Generation of Intentional Programs Within the Glial Syncytium

First of all, if one speaks of intentional programs, one has to define the formalism on which these programs are based.

### The Formalism of Negative Language

According to Guenther [16], a negative language can be formalized in an n-valent permutation system. Generally, a permutation of n things is defined as an ordered arrangement of all the members of the set taken all at a time according to the formula *n*! (! means factorial). Table 2 shows a quadrivalent permutation system in a lexicographic order. It consists of the integers 1, 2, 3, 4. The number of permutations is 24 (4! = 1.2.3.4 = 24).

**Table 2:** Quadrivalent (*n* = 4) permutation system arranged in a lexicographic order

	1	1	1	1	1	1	2	2	2	2	2	2	3	3	3	3	3	3	4	4	4	4	4	4
	2	2	3	3	4	4	1	1	3	3	4	4	1	1	2	2	4	4	1	1	2	2	3	3
	3	4	2	4	2	3	3	4	1	4	1	3	2	4	1	4	1	2	2	3	1	3	1	2
	4	3	4	2	3	2	4	3	4	1	3	1	4	2	4	1	2	1	3	2	3	1	2	1
Number of the permutation	1	2	3	4	5	6	7	8	9	10	11	12	13	14	15	16	17	18	19	20	21	22	23	24

This permutation system consists of 24 permutations (1 × 2 × 3 × 4…, 4 × 3 × 2 × 1) according to the formula *n* = 4! (factorial) = 1 × 2 × 3 × 4 = 24

The 24 permutations are lexicographically arranged

**Table:** 

1		4
2	to	3
3		2
4		1

The permutations of the elements can be generated with three different NOT operators N_1_, N_2_, N_3_ that exchange two adjacent (neighbored) integers (values) by the following scheme:

$$\begin{array}{*{20}c} 1 & \leftrightarrow & {2;} & 2 & \leftrightarrow & {3;} & 3 & \leftrightarrow & 4 \\ {} & {\left( {N_{1} } \right)} & {} & {} & {\left( {N_{2} } \right)} & {} & {} & {\left( {N_{3} } \right)} & {} \\ \end{array}$$

Generally, the number of negation operators (NOT) is dependent on the valuedness of the permutation system minus 1. For example, in a pentavalent permutation system four negation operators (*N*_1_, *N*_2_, *N*_3_, *N*_4_) (*n* = 5–1 = 4) are at work.

It is possible to form loops, each of which passes through all permutations of the permutation system once (Hamilton loop). In a quadrivalent system, they are computable (44 Hamilton loops), but in higher valent systems, they are not computable. Table 3 shows an example of a Hamilton loop [16]. The first permutation (*P* = 1234) is permutated via a sequence of negation operators (*N*_1.2.3…2.1.2_) generating all the permutations once until the loop is closed.

**Table 3:** Example of a Hamilton loop generated by a sequence of negative operators [16]

*p*	*N*	1.	2.	3.	2.	3.	2.	1.	2.	1.	2.	3.	2.	3.	2.	1	2.	1.	2.	3.	2.	3.	2.	1.	2.	*p*
1		2	3	4	4	3	2	1	1	2	3	4	4	3	2	1	1	2	3	4	4	3	2	1	1	
2		1	1	1	1	1	1	2	3	3	2	2	3	4	4	4	4	4	4	3	2	2	3	3	2	
3		3	2	2	3	4	4	4	4	4	4	3	2	2	3	2	1	1	1	1	1	1	1	2	3	
4		4	4	3	2	2	3	3	2	1	1	1	1	1	1	2	3	3	2	2	3	4	4	4	4	

The first permutation (*p* = 1 × 2 × 3 × 4) is permutated via a sequence of negation operators (*N*_1 × 2 × 3 × 4,….,2×1×2_) generating all the permutations once until the loop is closed (1234) in the sense of a Hamilton loop

**Fig. 1:**
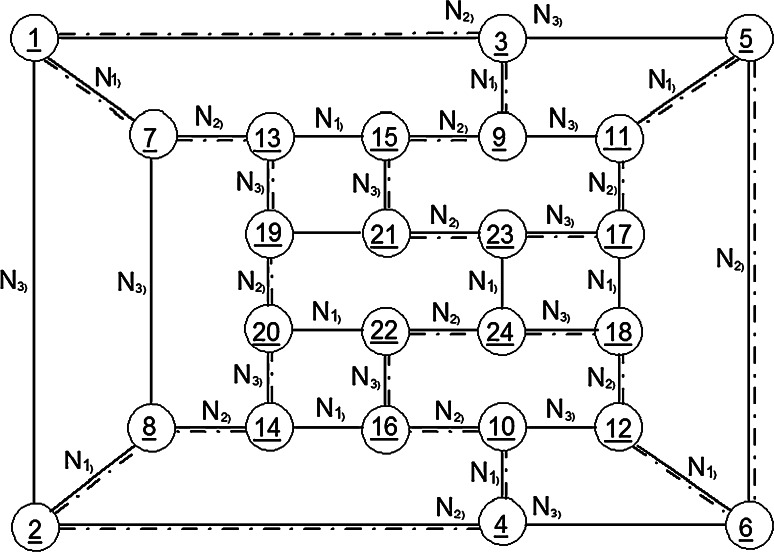
Example of a Hamilton loop in a quadrivalent permutograph. The numbers in *circles* represent the permutations (1,…,24) interconnected by negation operators( *N*_1_ …*N*_3_) of a closed permutation system called permutograph [15]. A Hamilton loop or negation sequence is indicated by a *dashed line*

Such permutation systems can be mathematically formalized as negation networks, called permutographs [17]. Figure 1 shows a quadrivalent permutograph. The individual NOT or negation functions N_1_, N_2_, N_3_ are represented between the permutations (1…24). The various Hamilton loops differ in NOT or negation operator sequence. An example of a Hamilton loop is indicated in this permutograph by a dash-dotted line. It is defined by the following negation operator sequence:

$$N_{1} {-}N_{2} {-}N_{3} {-}N_{2} {-}N_{3} {-}N_{2} {-}N_{1} {-}N_{2} {-}N_{1} {-}N_{2} {-}N_{3} {-}N_{2} {-}N_{3} {-}N_{2} {-}N_{1} {-}N_{2} {-}N_{1} {-}N_{2} {-}N_{3} {-}N_{2} {-}N_{3} {-}N_{2} {-}N_{1} {-}N_{2}$$

Already in the 1980s, it was shown that the negative language may represent an appropriate formal model for a description of intentional programs generated in neuronal networks of biological brains. Based on this formalism, computer systems for robot brains have also been proposed [10, 18]. Here, it is attempted to further elaborate on this possible intentional programming in our brains, focusing on glial–neuronal interaction.

### Glial Gap Junctions Could Embody Negation Operators

**Fig. 2:**
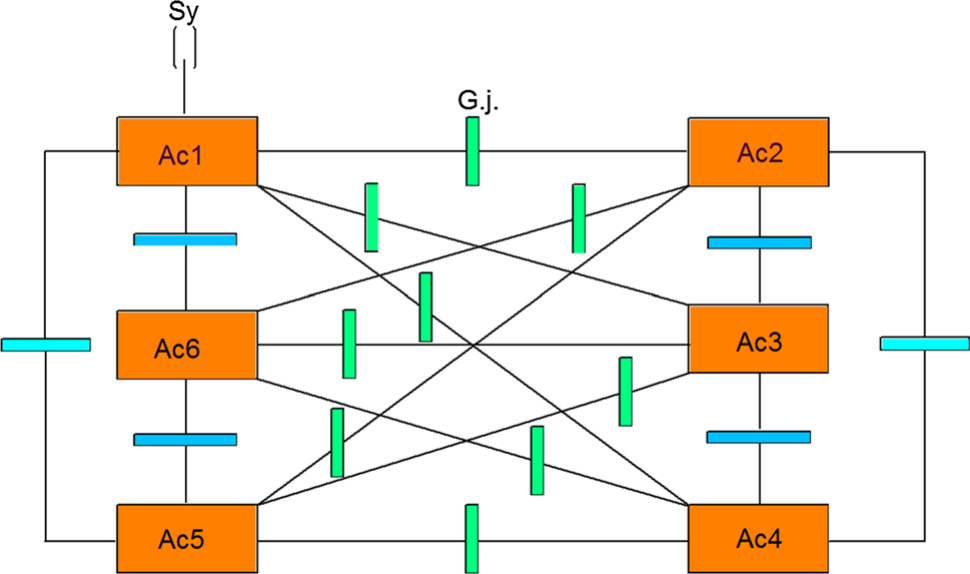
Outline of an astrocytic syncytium. Six astrocytes (A_c1_…A_c6_) are interconnected via 16 gap junctions (g.j.) building a complete syncytium. Each astrocyte contacts a neuronal synapse representing a tripartite synapse (for the sake of clarity, only one synaptic contact [Sy] is shown)

In situ morphological studies have shown that astrocyte gap junctions are localized between cell bodies, between processes and cell bodies, and between astrocytic end-feet that surround brain blood vessels. In vitro junctional coupling between astrocytes has also been observed (Fig. 2). Moreover, astrocyte-to-oligodendrocyte gap junctions have been identified between cell bodies, cell bodies, and processes, and between astrocyte processes and the outer myelin sheath. Thus, the astrocytic syncytium extends to oligodendrocytes, allowing glial cells to form a generalized glial syncytium, also called “panglial syncytium”, a large glial network that extends radially from the spinal cord and brain ventricles, across gray and white matter regions, to the glia limitans and to the capillary epithelium.

Ependymal cells are also part of the panglial syncytium. Additionally, activated microglia may also be interconnected with astrocytes via gap junctions. However, the astrocyte is the linchpin of the panglial syncytium. It is the only cell that interconnects to all other glia. Furthermore, it is the only one with perisynaptic processes.

Gap junctions are channels that link the cytoplasm of adjacent cells and permit the intercellular exchange of small molecules with a molecular mass <1–1.4 kDa, including ions, metabolites, and second messengers. IP3 is the most important since this initiates the calcium wave in the attached cell after it transverses the gap junction channel [19]. In addition to homologous coupling between cells of the same general class, heterologous coupling has been observed between astrocytes and oligodendrocytes. Newman [20] has demonstrated that gap junctions interconnect Muller cell to Muller cell and Muller cell to regular astrocytes in the retina. Homologous and heterologous coupling could serve to synchronize the activities of neighboring cells that serve the same functions. Such coupling could extend the size of a functional compartment from a single cell to a multi-cellular syncytium, acting as a functional network.

Gap junctions are now recognized as a diverse group of channels that vary in their permeability, voltage sensitivities, and potential for modulation by intracellular factors; thus, heterotypic coupling may also serve to coordinate the activities of the coupled cells by providing a pathway for the selective exchange of molecules below a certain size. In addition, some gap junctions are chemically rectifying, favoring the transfer of certain molecules in one direction versus the opposite direction. The main gap junction protein of astrocytes is connexin (Cx) 43, whereas Cx32 is expressed in oligodendrocytes in the CSN as well as another type of connexin, Cx45. Heterologous astro-oligodendrocyte gap junctions may be composed of Cx43/Cx32, if these connexins form functional junctions [21]. Recent experimental results suggest roles of glial gap junction-mediated anchoring of signaling molecules in a wide variety of glial homeostatic processes [22].

Gap junctions are showing properties that differ significantly from chemical synapses [23–25]. The following enumeration of gap junctional properties in glial syncytia may support the hypothesis that gap junctions could embody negation operators in the sense of a generation of negative language in glial syncytia:

First, gap junctions communicate through ion currents in a bidirectional manner, comparable to negation operators defined as exchange relations. Bidirectional information occurs between astrocytes and neurons at the synapse. This is primarily chemical and based on neurotransmitters. It is not certain that all glial gap junction communications are bidirectional due to rectification. This is a poorly understood area because of extremely severe technical difficulties, especially in vivo [26]. Second, differential levels of connexin expression reflect region-to-region differences in functional requirements for different astrocytic gap junctional coupling states. The presence of several connexins enables different permeabilities to ions and molecules and different conductance regulation. Such differences of gap junctional functions could correspond to the different types of negation operators. Third, neuronal gap junctions do not form syncytia and are generally restricted to one synapse. Fourth, processing within a syncytium is driven by neuronal input and depends on normal neuronal functioning. The two systems are indivisible. It is important to emphasize that neuronal activity-dependent gap junctional communication in the astrocytic syncytium is long-term potentiated. This is indicative of a memory system as proposed in neuronal synaptic activity by Hebb over six decades ago [27]. Fifth, the diversity of astrocytic gap junctions results in complex forms of intercellular communication because of the complex rectification between such numerous combinatorial possibilities. Sixth, the astrocytic system may normally function to induce precise efferent (e.g., behaviorally intentional or appropriate motor) neuronal responses. Admittedly, the testing of this conjecture is also faced with experimental difficulties. Since gap junctional plaques play a central role in glial networks, let me describe some further details. Electrophysiological analysis of the rate at which functional gap junctional channels accumulate at cell–cell interfaces indicates that plaque formation is a cooperative self-assembly process [28]. Connexin protein has a half life of only 1,5 to 3,5 h. Because gap junction assembly appears to be a cooperative self-assembly process, reducing the rate of connexin degradation would lead to a large increase in gap junction formation and intercellular communication [29]. Most importantly, it has been hypothesized that a high turnover rate in combination with a low percentage of functional channels (about 10 % in a plaque) coupling [29] may enable this relative number of cells to compute circles serving as intentional programs.

Now, let us tie gap junctional functions and negative language together. Negation operators represent exchange relations between adjacent values or numbers. So they operate like gap junctions bidirectionally. Dependent on the number of values (*n*) that constitute a permutation system, the operation of different negation operators (*n*−1) is necessary for the generation of a negative language. With concern to gap junctions, they also show functional differences basically influenced by the connexins. Therefore, different types of gap junctions could embody different types of negation operators. Furthermore, a permutation system represents—like the glial syncytium—a closed network generating a negative language. So we have a biomimetic interpretation of the negative language. But what makes that language so intentional?

### Glial Generation of Cyclic Pathways in Neuronal Networks

**Fig. 3:**
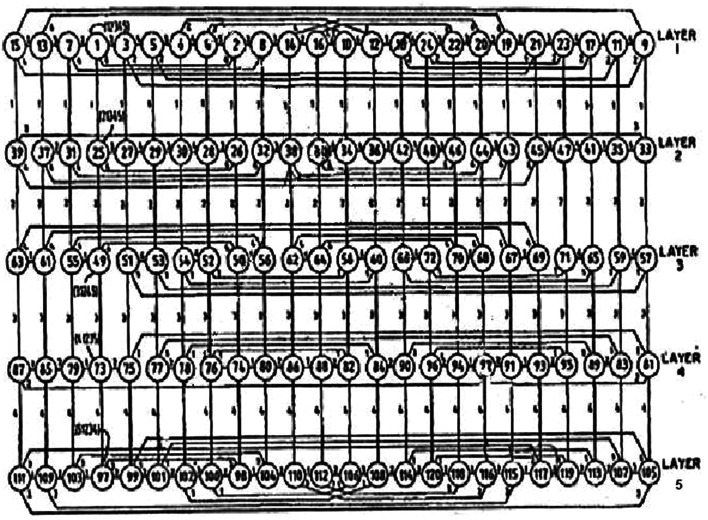
Example of a pentavalent (*n* = 5) permutograph arranged in layers. For *n* = 5, i.e., for a pentavalent logic, a schematic circuit diagram of a permutation system (permutograph) is shown. The 120 permutations (according to the formula *n*! = 5! = 1 × 2 × 3 × 4 × 5 = 120) are shown as *circles*. The individual permutations are numbered consecutively from 1 to 120, *each number* representing one of the 120 permutations. They are interconnected by (*n*−1) negation operators (*N*_1_–*N*_4_). For example, permutation (1) stated in the upper layer 1 should be read “1 2 3 4 5”. This permutation may be converted into permutation (7) corresponding to “1 3 2 4 5” by applying the negator *N*_2_, i.e., by exchanging the values 2 and 3. The negation operators, i.e., exchange operations of successive numerical values within permutations, are shown in this figure by the smaller numerical values [17]

Now we are confronted with the question what part of the permutation system proposed could be embodied by the neuronal network. It is hypothesized that the neuronal network could embody the permutations of a permutographic system. For example, a quadrivalent permutation system may be interpreted as a neuronal network. In Table 3, only the 24 permutations (1234,…,4321) are shown. Each permutation formalizes a neuron with a specific computational quality. In parallel, the permutations determine how neurons can be interconnected according to the rule of manyvalent negation operators (*N*_1_, *N*_2_, *N*_3_) building a neuronal network that embodies a permutation system. Figure 3 shows an example of a pentavalent permutograph [18]. The numbers in circles designate the permutations (*n* = 5! = 120). The interconnecting lines represent negation operators (1, 2, 3, 4).

As already supposed, the glial syncytium could compute various sequences of negation operators in order to test their feasibility in the neuronal permutographic network. This is similar to a kind of intentional pathfinding in neuronal networks. From a biocybernetic point of view, living systems are self-referring systems [30]. On the highest level, they are capable of self-reflection or self-observation. Formally speaking, our brain is permanently generating such reflection cycles. A cycle is not hierarchically ordered, but follows the rule of heterarchy (A-B-C-D-A) [31]. Therefore, the pathfinding of glial intentional programs in neuronal networks is only successful if it results in a closed pathway in form of a cycle. In the case of a cycle that passes all neurons once in the network, we speak of a Hamilton loop. Such loops may occur in the neuronal system associated with gap junctions of the glial syncytium.

With concern to the realization of glial intentional programs, there are several possibilities. First, a sequence of negation operators is erroneous, since it is unable to find a cycle. Second, a successful finding of a cycle is not reinforced by appropriate sensory information, so that the intentional program is unfeasible with regard to the environment. Third, a cycle generated by a glial intentional program corresponds to a neuronal network that is activated by sensory information. Fourth, humans are able to reject a feasible intentional program, since another program has priority for a period of time. Here, one can see a parallel to Edelman’s “Neural Darwinism” [32]. He proposed a multi-draft hypothesis where several intentional possibilities are generated, but only the one with the best response is actually generated. Fifth, the possible cyclic pathways in superastronomic complex neuronal networks offer glial intentional programs the chance to find new cyclic pathways in the sense of creativity. In other words, the neuronal system is interpreting the intentional possibilities generated in the astrocytic syncytium. Sixth, supposing that the glial syncytium also has a memory function similar to the neuronal system [33], it could “self-imprint” already successful intentional programs in the syncytium, which implies a form of learning. This has been experimentally verified by Pasti et al. [34] who showed that calcium waves in the glial syncytium undergo a form of long-term potentiation based on neuronal activation.

Experimentally verified knowledge of glial–neuronal interaction may – at least partly – support this hypothetical model of intentional glial–neuronal interaction. First of all, the communication between astrocytes and neurons occurs bidirectionally [35]. Additionally, a bidirectional feedback between astrocytes and neurons at each synapse results in the coding and integration of calcium waves, as they travel through the glial syncytium. Therefore, each perisynaptic astrocytic filopodal process (several may be present at each synapse) is a member of the syncytium. This gives a huge global distribution form of information processing throughout the brain [26].

Most important to the proposed model of glial–neuronal interaction are experimental findings concerning synaptic activation of astrocytes evoking feedback neuronal synchronization [36]. These researchers observed in hippocampal slices how two or more slow inward currents recorded in the same neuron can have strikingly different kinetics suggesting the presence of multiple release sites from either one or many astrocytes impinging onto an individual neuron. By cooperating with the excitatory synaptic inputs to recruit specific subsets of neurons in the neuronal network, the activation of extrasynaptic NMDA receptors by astrocytic glutamate may represent a flexible mechanism that favors the formation of dynamically associated assemblies of neurons. In fact, glial intentional programs could operate in neuronal networks based on such mechanisms. In other words, successful glial pathfindings in neuronal networks could be interpreted as the formation of dynamically associated assemblies of neurons. Additionally, the glial syncytium is self-organized [37]. Most importantly, one astrocyte can establish through its filopodal processes contact with approximately 145.000 synapses, each of which acts as a subcellular microdomain for information processing via calcium signaling and bidirectional feedback [38]. Additionally, each microdomain independently responds to various combinations of neurotransmitter signals. This occurs at low neuronal activation. Intracellular calcium signals with associated intercellular syncytial transfer of information occur with increasing neuronal synaptic activation [39]. But the possible memory-based learning effect in glial syncytia is extremely difficult to study.

However, the role of gap junctions in memory formation can be interpreted as follows: Gap junctions could register already generated cyclic pathways in the syncytium (formalized as a sequence of negation operators). Depending on a positive feedback from the neuronal network to the glial syncytium based on feasible intentions in regard to environmental information, gap junctions could strengthen their structure embodying a memory mechanism. If that would be the case, then we have a double memory function of gap junctions: a local embodiment of memories, on the one hand, and a pathway memory determined by gap junctions, on the other hand. This has already been experimentally verified [40].

At this point, one could argue that neuronal mechanisms per se may compute intentional behavior, so that it is not necessary to refer to the glial syncytium. For example, mirror neurons are premotor neurons that fire when the subject performs an object-directed action, and they also fire when the subject observes someone else performing the same class of actions. Because action implies a goal, it has been proposed that mirror neurons provide a neural mechanism for understanding the intentions of others [41]. However, here we deal with the neural computation of intentions of others, and not how intentions may be generated in the brain per se. Note that only the latter problem is the topic of the present paper which hypothesizes that the glial syncytium may play a decisive role.

## Embodiment of Hamilton Loops in Glial Gap Junctional Plaques

The underlying formalism has already been described. It is assumed that each glial gap junctional plaque embodying a Hamilton loop value is excited in the neuronal system (n-plaque chips) dependant on the environmental information computed by the perception systems. In Table 4, a so-called Guenther matrix is computed consisting of 24 Hamilton loops. Formally, it can be shown that it is possible to start on any location of a 4-valued permutation system to generate a Hamilton loop [42].

**Table 4:** Günther matrix consisting of 24 Hamilton loops

Permutations	1	1	1	1	1	1	2	2	2	2	2	2	3	3	3	3	3	3	4	4	4	4	4	4
	2	2	3	3	4	4	1	1	3	3	4	4	1	1	2	2	4	4	1	1	2	2	3	3
	3	4	2	4	2	3	3	4	1	4	1	3	2	4	1	4	1	2	2	3	1	3	1	2
	4	3	4	2	3	2	4	3	4	1	3	1	4	2	4	1	2	1	3	2	3	1	2	1
Number of Permutations	1	2	3	4	5	6	7	8	9	10	11	12	13	14	15	16	17	18	19	20	21	22	23	24
HL																								
Hamilton loop 1	1	8	24	9	17	16	2	7	23	10	16	15	3	6	22	11	19	14	4	5	21	12	20	13
Hamilton loop 2	24	1	17	8	16	9	23	2	18	7	15	10	22	3	19	6	14	11	21	4	20	5	13	12
Hamilton loop 3	24	17	1	16	8	9	23	18	2	15	7	10	22	19	3	14	6	11	21	20	4	13	5	12
Hamilton loop 4	17	24	16	1	9	5	18	22	15	2	10	7	19	22	14	3	11	6	20	21	13	4	12	5
Hamilton loop 5	17	16	24	9	1	9	18	15	23	10	2	7	19	14	22	11	3	6	20	13	21	12	4	5
Hamilton loop 6	16	17	9	24	8	1	15	18	10	23	7	2	14	19	11	22	6	3	13	20	12	21	5	4
Hamilton loop 7	24	7	23	8	16	15	1	6	22	9	17	14	2	5	21	10	18	13	3	4	20	11	19	12
Hamilton loop 8	23	24	16	7	15	8	22	1	17	6	14	9	21	2	18	5	13	10	20	3	19	4	12	11
Hamilton loop 9	23	16	24	15	7	8	22	17	1	14	6	9	21	18	2	13	5	10	20	19	3	12	4	11
Hamilton loop 10	16	23	15	24	8	7	17	22	14	1	9	6	18	21	13	2	10	5	19	20	12	3	11	4
Hamilton loop 11	16	15	23	8	24	7	17	14	22	9	1	6	18	13	21	10	2	5	19	12	20	11	3	4
Hamilton loop 12	15	16	8	23	7	24	14	19	9	22	6	1	13	18	10	21	5	2	12	19	11	20	4	3
Hamilton loop 13	23	22	6	15	7	14	24	21	5	16	8	13	1	20	4	17	9	12	2	19	3	18	10	11
Hamilton loop 14	22	23	15	6	14	7	21	24	16	5	13	8	20	1	17	4	12	9	19	2	18	9	11	10
Hamilton loop 15	22	15	23	14	6	7	21	16	24	13	5	8	20	17	1	12	4	9	19	18	2	11	3	10
Hamilton loop 16	15	22	14	23	7	6	16	21	13	24	8	5	17	20	12	1	9	4	18	19	11	2	10	3
Hamilton loop 17	15	14	22	7	23	6	16	13	21	8	24	5	17	12	20	9	1	4	18	11	19	10	2	3
Hamilton loop 18	14	15	7	22	6	23	13	16	8	21	5	24	12	17	9	20	4	1	11	18	10	19	3	2
Hamilton loop 19	22	5	21	6	14	13	23	4	20	7	15	12	24	3	19	8	16	11	1	2	18	9	17	10
Hamilton loop 20	5	22	6	21	13	14	4	23	7	20	12	15	3	24	6	19	11	16	2	1	9	18	10	17
Hamilton loop 21	5	6	22	13	21	14	4	7	23	12	20	15	3	8	24	11	19	16	2	9	1	10	18	17
Hamilton loop 22	14	21	13	22	6	5	15	20	12	23	7	4	16	19	11	24	8	3	17	18	10	1	9	2
Hamilton loop 23	14	13	21	6	22	5	15	12	20	7	23	4	16	11	19	8	24	3	17	10	18	9	1	2
Hamilton loop 24	13	14	6	21	5	22	12	15	7	20	4	23	11	16	8	19	3	24	10	17	9	18	2	1

The permutation where the counting starts is stepwise displaced from the extreme left to the extreme right. However, one can start on every permutation. The matrix shows 24 Hamilton loops

**Fig. 4:**
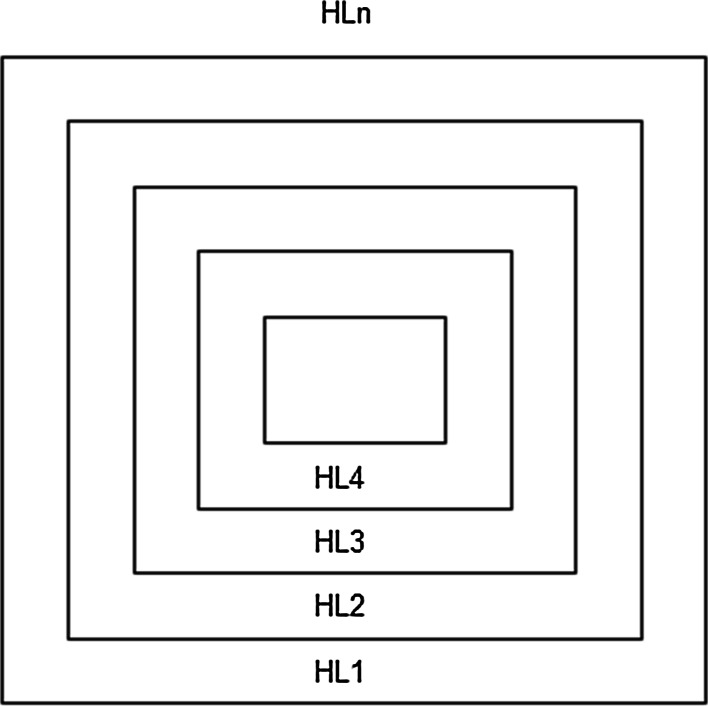
Intentional programs embodied by gap junctions building a plaque. Four Hamilton loops (HL_1_ …HL_4_) building a gap junctional plaque consisting of n-Hamilton loops (HL_*n*_) are depicted as described in the text. Each Hamilton loop represents an intentional program. Geometrically, a gap junctional plaque is drawn in *squares*

Figure 4 depicts a plaque which embodies all Hamilton loops (drawn as squares; for sake of clarity only 4 squares are shown). Note that McCulloch interpreted the modes of behavior as pairs of opposites, for example wakefulness–sleeping or eating—to void (urinate—defecate). Formally speaking, this affords a glial gap junctional network of 88 Hamilton loops consisting of 44 loops in one direction and 44 in the opposite direction. From the biology of the neuronal system, we know that gap junctional plaques decay within a time span of hours (about 4 h) and then reorganize again. It may be important that the embodiment of Hamilton loops is redundant. We assume that modes of behavior necessary for the maintenance of the living organism (like eating) are manifoldly recorded such that a plaque structure consists not only of about 24 Hamilton loops but also of 44, as formally computed. The same may hold for the Hamilton loop with a reverse run. In this manner, each Hamilton loop embodies a structure of 88 Hamilton loops.

## Outline of the Implementation of the Reticular Formation in a Robot Brain

**Fig. 5:**
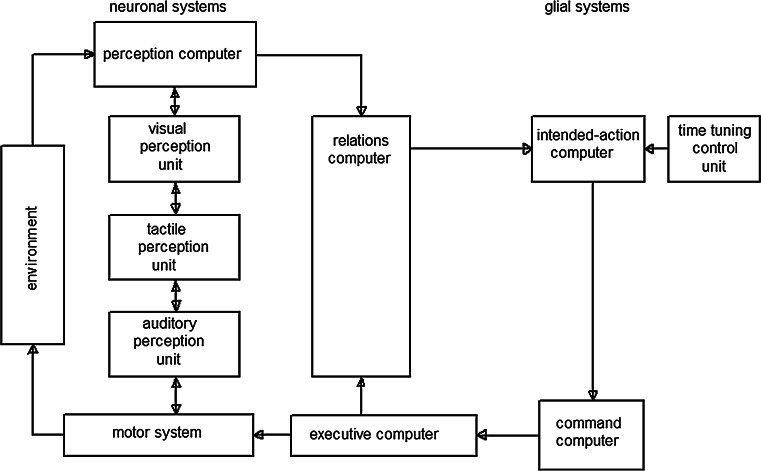
Computer system for simulating the reticular formation based on glial–neuronal interactions. The neuronal system essentially consists of the perception computer integrated by a relation computer [44] and an executive computer for the motor system executing the result of the neuronal and glial system in the environment. The glial network is implemented as an intended action computer system and a timing control unit. The computed intentional programs are transferred to a command computer which commands which intentional program is selected for the executive computer

I have already simulated a computer system for the neuronal networks of the reticular formation of the brainstem [10]. Here, the glial networks of the reticular formation are additionally outlined. Accordingly, a system for the simulation of the whole reticular formation is described. The system is comprised of a central processing unit, a command computer structured on the basis of a permutograph with a plurality of storage modules [10], with the storage modules corresponding to the elements, and the connection between the storage modules to the edges of the permutograph (not shown in Fig. 5). The connections establish internal circuits which correspond to the negation sequences of the permutograph in the form of Hamilton loops, each of which is associated with a behavior pattern of the reticular formation. The command computer is controlled by input computers in which a preprogrammed intended action is related to environmental information. The relation computer integrates the different types of perception systems [43]. Originally, the command computer has been positioned in the neuronal network, but this seems not to be necessary if one attributes the generation of intentional programs to the glial network or to glial gap junctional plaques. Hence, in the neuronal network, only an executive computer is at work to execute a mode of behavior (Fig. 5).

## The Integrative Function of the Reticular Formation

Since the reticular formation is interconnected with all other brain regions, especially the limbic system and the cerebral cortex, it is able to integrate its generated action programs with the actual information of the perception and motor systems [45]. Let me give the example of the action modes “look,” “forward,” “stop,” and “retreat.” This program sequence is established by a storage module associated with Hamilton loop HL1 to HL4. This run is monitored by the timing control unit. During the program run the perception computer and the relation computer (Fig. 5) constantly provide new information which is compared with the intended actions, in the following manner: Suppose that during the execution of the intended action program, the perception system detects an obstacle. An object stands in the way (program 3), so it is necessary to retreat (program 4). Then look for a new path (program 1) and move forward (program 2). If following weighting in the relation computer this new program sequence is identified as having priority, the relation computer interrupts the command execution in the command computer and switches the latter to the new program sequence (for technical details, see [10] ).

## Conclusion

A model is proposed based on glial–neuronal interactions in the reticular formation of the brainstem. Formally, a new logic of relations called permutograph is applied. This graph-theoretical formalism uses exchange relations between neighboring values. This model may enable the implementation in a robot brain, as outlined above. The original simulation of the neuronal system in the reticular formation is further elaborated for glial networks. The networks build gap junctional plaques that may embody n-Hamilton circles, each of which represents a mode of behavior generated in the glial system and executed in the neuronal system of the reticular formation. In this way, the whole body could execute various integrative behaviors.

Admittedly, the glial network of the reticular formation has as yet not been experimentally identified in brain research, although pertinent technical progress is promising. However, robotics may offer a real alternative. If we implement the model proposed here in a robot brain, it should be able to produce different modes of behavior. In this way, we could learn if we are right or wrong. Since intentional programming is an essential feature of living systems, such robots may also show a “touch of subjectivity.”
